# The implementation of unit-based perinatal mortality audit in perinatal cooperation units in the northern region of the Netherlands

**DOI:** 10.1186/1472-6963-12-195

**Published:** 2012-07-09

**Authors:** Mariet Th van Diem, Albertus Timmer, Klasien A Bergman, Katelijne Bouman, Nico van Egmond, Dennis A Stant, Lida H M Ulkeman, Wenda B Veen, JanJaapHM Erwich

**Affiliations:** 1 Department of Obstetrics and Gynaecology, CB22, University Medical Centre Groningen, P.O. Box 30001, 9700 RB, Groningen, The Netherlands; 2 Department of Pathology, University Medical Centre Groningen, Groningen, The Netherlands; 3 Department of Neonatology, University Medical Centre Groningen, Groningen, The Netherlands; 4 Department of Genetics, University Medical Centre Groningen, Groningen, The Netherlands; 5 General Practitioners Practice “De Kompe”, Gorredijk, The Netherlands; 6 Department of Epidemiology, University Medical Centre Groningen, Groningen, The Netherlands; 7 Legal Department, University Medical Centre Groningen, Groningen, The Netherlands

## Abstract

**Background:**

Perinatal (mortality) audit can be considered to be a way to improve the careprocess for all pregnant women and their newborns by creating an opportunity to learn from unwanted events in the care process. In unit-based perinatal audit, the caregivers involved in cases that result in mortality are usually part of the audit group. This makes such an audit a delicate matter.

**Methods:**

The purpose of this study was to implement unit-based perinatal mortality audit in all 15 perinatal cooperation units in the northern region of the Netherlands between September 2007 and March 2010. These units consist of hospital-based and independent community-based perinatal caregivers. The implementation strategy encompassed an information plan, an organization plan, and a training plan. The main outcomes are the number of participating perinatal cooperation units at the end of the project, the identified substandard factors (SSF), the actions to improve care, and the opinions of the participants.

**Results:**

The perinatal mortality audit was implemented in all 15 perinatal cooperation units. 677 different caregivers analyzed 112 cases of perinatal mortality and identified 163 substandard factors. In 31% of cases the guidelines were not followed and in 23% care was not according to normal practice. In 28% of cases, the documentation was not in order, while in 13% of cases the communication between caregivers was insufficient. 442 actions to improve care were reported for ‘external cooperation’ (15%), ‘internal cooperation’ (17%), ‘practice organization’ (26%), ‘training and education’ (10%), and ‘medical performance’ (27%). Valued aspects of the audit meetings were: the multidisciplinary character (13%), the collective and non-judgmental search for substandard factors (21%), the perception of safety (13%), the motivation to reflect on one’s own professional performance (5%), and the inherent postgraduate education (10%).

**Conclusion:**

Following our implementation strategy, the perinatal mortality audit has been successfully implemented in all 15 perinatal cooperation units. An important feature was our emphasis on the delicate character of the caregivers evaluating the care they provided. However, the actual implementation of the proposed actions for improving care is still a point of concern.

## Background

Perinatal audit is defined as the systematic and critical analysis of the quality of medical care, including the procedures used for diagnosis and treatment, the use of resources, and the resulting outcome and quality of life for mother and child [1,2]. Perinatal mortality audit can be considered to be a way to improve the care process for all pregnant women and their newborns [3,4]. It creates an opportunity to learn from unwanted events in the care process by identifying and analyzing these events and subsequently taking steps to prevent them from occurring again [5].

Perinatal audit is performed at different levels, using different methods with different primary objectives [6,7]. In an external audit, the care process is evaluated by independent external auditors, followed by feedback to the caregivers involved. A unit-based audit is usually an internal audit in which caregivers evaluate their own care and have the opportunity to take immediate, fast measures to improve care. An internal audit with the caregivers involved in the case and their other colleagues is a delicate matter. Factors, such as interpersonal (hierarchical) relationships and competition issues, may influence the analysis. However, with an internal audit conducted by the local groups themselves, more essential details can be expected to be revealed, which in turn can lead to more efficient improvements at a local level.

The Dutch perinatal care system is unique with respect to its community-based care, including home deliveries. Inherent to this system is that when a change in the pregnancy risk profile occurs, the woman may be referred from community- to hospital-based care, and vice versa, during the pregnancy or the delivery. This poses not only challenges for the care provided, but also for the evaluation of this care by means of a perinatal audit. Firstly, audit groups need to be fairly large in order to have representatives, and thus expertise, of obstetric and neonatal care at all levels (obstetrician, perinatologist, general practitioner, independent and hospital midwives, obstetric and pediatric nurses, pediatrician, neonatologist, pathologist and geneticist). Secondly, the care process is inherently complicated by the many handover moments, and above all, information on the care process must be obtained from several caregivers both in the community and from one or more hospitals.

Implementing changes, such as introducing a perinatal audit, is a process with several stages, ranging from creating awareness, implementation, integration to sustainment.[8]

In this article we report on the results of the implementation process of unit-based perinatal audit in 15 perinatal cooperation units in the northern region of the Netherlands. We focus on the implementation strategy of these meetings, the number of participating perinatal cooperation units at the end of the project, the identified substandard factors (SSF), the actions to improve the care as a result of the meetings, and the opinions of the participants.

Ethics approval was not required for implementation studies on the quality of care which did not involve experiments on humans.

## Methods

### *Project team and confidential committee*

The project team consisted of representatives of all perinatal caregiver groups, a methodologist, a legal advisor, and a psychologist. The team participated in an introductory training in all 15 audit groups. At least two team members attended the following audit meetings. The project team provided an independent chairperson for the first and following audit meetings until other independent chairpersons were found. As a team member, the project coordinator supported the unit-based core groups in organizing and preparing the audit meetings.

A confidential committee was installed in view of the delicate character of these audits, in which caregivers reflect on the care they provided within their own unit. The committee operated independently from the project team and the members were from outside the northern region. They were asked to assist caregivers in the case of psychological or procedural complaints emerging from the audit meetings.

### *Area and participants*

Unit-based perinatal audit was introduced in all 15 perinatal cooperation units in the northern region of the Netherlands from September 2007 till March 2010. A cooperation unit consists of hospital-based perinatal caregivers and community-based perinatal caregivers in the hospital’s catchment area. In each unit, a multidisciplinary core group was formed to organize and prepare the audit meetings. The unit-based audit group is a larger multidisciplinary group consisting of all the perinatal caregivers, including gynecologists, pediatricians, hospital and independent midwives, obstetric and pediatric nurses, general practitioners, pathologists and a clinical geneticist, working in the hospital and in its catchment area.

### *Implementation strategy*

Awareness was first created by informing all perinatal caregivers and the chairmen of the hospital boards in the study area by sending a personal letter in which the project was introduced. Prior to finalizing the implementation strategy, the project coordinator held semi-structured interviews with key figures of the caregiver groups and the hospital management boards to gain insight into factors likely to impede or stimulate the implementation process and to create more awareness for the concept of a perinatal audit.

**Figure 1:**
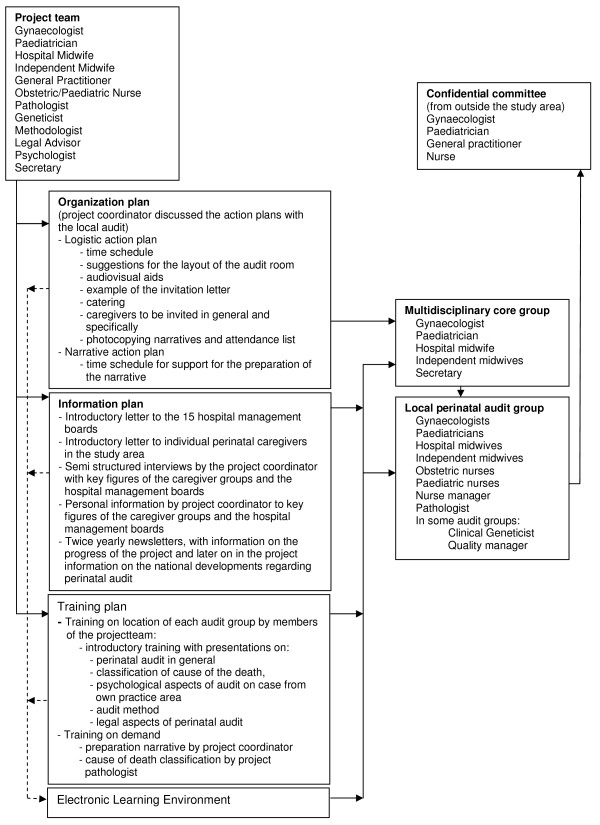
The implementation strategy for local perinatal audit meetings.

The strategy also consisted of an information plan, an organization plan, and a training plan. Figure 1 shows the contents of these plans and the relations to the project team, the confidential committee, the core groups, and the audit groups (Figure 1).

### *Perinatal mortality cases*

Perinatal mortality cases were defined as cases with fetal or neonatal mortality with a gestational age of > 22 weeks and/or a crown/heel length of 25 cm and/or a birth weight of  > 500 grams up to 28 days after birth [9,10]. Cases were selected by the core groups of the perinatal cooperation units, as they occurred or, in the larger units, for their potential to identify areas to improve care.

### *Narrative*

Anonymous narratives were the basic documents used in the perinatal audit meetings. The care process and the circumstances under which the mortality occurred were outlined in a set narrative format. In addition, using the when-what-why concept, the cause of death was classified by the local core group and added to the narrative [11]. The caregivers involved in the case prepared these narratives or were consulted when other members of the core group prepared the narrative.

### *Substandard Factors (SSF)*

SSF was defined as a care management problem involving care that deviated from the safe limits of practice as laid down in guidelines, standards, protocols or normal practice, and that had the potential to lead, directly or indirectly, to an adverse outcome for the patient [12].

### *Audit instrument*

The audit instrument was based on a root cause analysis. The most distinctive feature of a root cause analysis is the fundamental idea that the occurrence of a SSF is seldom attributable to just one person, but is usually the result of a variety of underlying causes [13,14]. This instrument was introduced in the unit-based perinatal audit groups as the “6 What Questions". By answering these 6 questions, the SSF could be analyzed, making it possible to identify the origin of the SSF, the caregiver groups involved, to draw conclusions, and to formulate the actions needed to prevent the SSF from occurring again (Table 1) [15,16]. 

**Table 1:** The 6 “What” questions for the analysis of substandard factors*

**What question**	**Question and sub questions (underlying purpose of the question or response options)**
1.	**what happened?***(identified substandard factor to be analysed)* Stating the identified substandard factor defined as the care management problem that involves care that deviates from safe limits of practice as laid down in guidelines etc and had the potential to lead, directly or indirectly, to an adverse outcome for the patient
	1a**. which caregivers were involved** Here all caregivers, including secretarial, paramedical and auxiliary staff involved in the occurrence of the substandard factor are listed.
2.	**what were the circumstances in which the ssf occurred?***(description of the situation)* Short description of the relevant circumstances in which the SSF occurred (e.g. salient clinical events, timeframe, weekend or workday, physical and mental state of the patient, local situation, workload of the care giver, etc)
3.	**what made the ssf occur?** (*analyses of underlying causes*)
	Stating the underlying cause(s) for the occurrence of the SSF, categorized into 6 groups:
	- patient related (e.g. distress, seriousness of the condition),
	- task related (e.g. availability of protocols and laboratory facilities),
	- care giver related (e.g. motivation, attitude, skills),
	- team related (e.g. communication between care givers, availability of supervision)
	- work environment related (e.g. staffing mix, availability of supporting staff)
	- management related (safety culture, financial resources)
	3a. determination if the underlying causes are relevant only to the case under analysis or a structural problem in the organization
4.	**what is the relation between the ssf and death?***(categorization of the relation)*
	- none: there is no relation between the identified SSF and the outcome
	- unlikely: it is unlikely that different management would have made a difference to the outcome
	- possible: different management might have made a difference to the outcome
	- probable: different management would reasonably be expected to have made a difference to the outcome.
	- very probable: a clearly avoidable factor implying that the adverse outcome could have been prevented.
5.	**what are the conclusions?***(analysis of the answers 1-4)* Gives a point by point list of underlying causes leading to the occurrence of the SSF. (e.g. inadequate supervision, failure of monitoring equipment)
6.	**what needs to be done to prevent the ssf from occurring again?***(action points)* Gives a point to point list of action points for the improvement of care. (e.g. make a skills and drills program (including a roster) for all relevant personnel)

* Based on the work of Vincent and Young (Vincent 2003, Young 2001).

### *Audit*

Using the narrative, SSF were identified during unit-based perinatal audit meetings. Members of the project team chaired these meetings until other independent chairpersons were found. In order to create and sustain a safe and secure environment, three fundamental rules were formulated and used at every meeting: (1) “the meeting is confidential”, (2) “all caregivers are considered to be ‘experts’ in their own professional area”, and (3) “instead of judging each other, caregivers ask each other inquisitive and non-judgmental questions”. Using the 6 “What” questions format, the identified SSF could be systematically analyzed with the entire audit group or in smaller groups, and the action points needed to improve the care could be formulated.

### *Data collection*

Attendance lists were used to collect data on attendance by the invited caregivers. A specially designed questionnaire was used to collect data on the participants’ opinion of the unit-based audit meeting as a whole, on their perception of security within the group, the opportunity for each member to take part in the discussion, the educational aspect of perinatal audit, and the changes the caregivers implemented as a result of the audit meeting.

### *Data analyses*

Data on SSF were categorized into ‘use of guidelines’, ‘content of guidelines’, ‘normal practice’, ‘communication’, ‘documentation’ and ‘medication and investigations’, which were divided into further subcategories matching the main categories. The actions after audit meetings were categorized into ‘external cooperation’, ‘internal cooperation’, ‘organization of practice’, ‘training and education’, ‘medical’ and other. SPSS for Windows version 16.0 was used to calculate frequencies.

## Results

### *Area and participants*

Between September 2007 and March 2010, unit-based perinatal audit was introduced in 15 perinatal cooperation units in the northern region of the Netherlands. At the end of the project, two hospitals with their respective cooperation units merged, but all the cooperation units continued to perform perinatal audit meetings using the methods that were introduced.

### *Audit meetings*

In total 64 unit-based audit meetings were held. These were plenary audit meetings held twice yearly (49), to which all perinatal caregivers of the cooperation unit were invited. Because of the large number of cases in one university centre and in one large regional hospital, the frequency of the audit meetings in these centers was changed halfway though the project to monthly (13) and three-monthly (2) meetings, respectively, with smaller audit groups. These smaller groups, which also included one representative of each perinatal caregiver group and the caregivers involved in the case, discussed these cases. Apart from the smaller audit groups and one large audit group, all perinatal audit meetings were organized outside office hours.

### *Participants and cases*

In total, 245 midwives (hospital-based and independent), 103 nurses (obstetric and pediatric), 100 obstetricians (including registrars and house officers), 64 pediatricians (including registrars and house officers), 11 pathologists, 3 clinical geneticists, 53 general practitioners (including registrars), 48 students (medicine and midwifery), 16 managerial staff (department, sector, higher), 16 secretarial staff, and 18 other staff (e.g. quality management) participated in the audit meetings one or more times. In total there were 677 particiants and they audited 112 cases of perinatal mortality. Table 2 lists the cases by gestational age at birth and by time of death in relation to the birth.

**Table 2:** 111* audited cases described by gestational age at birth and period of death

	Antepartum death n(%)*		**Intrapartum death n(%)**		**Neonatal death < 24 hrs n(%)**		**Neonatal death 24 hrs-1 wk n(%)**		**Neonatal death 1 wk-28 days n(%)**		**Total n(%)**	
**Gestational age**												
22- 23^6^ wks	3	(4)	-		6	(38)	-	-	-	-	9	(8)
24-27^6^ wks	3	(4)	-		-	-	-	-	2	(22)	5	(5)
28-31^6^ wks	11	(16)	-		1	(6)	1	(10)	-	-	13	(12)
32-36^6^ wks	15	(22)	1	(13)	1	(6)	2	(20)	1	(11)	20	(18)
37-40^6^ wks	29	(43)	5	(63)	4	(25)	5	(50)	6	(67)	49	(45)
>41 wks	7	(10)	2	(25)	4	(25)	2	(20)	-	-	15	(13)

* one case survived, but was audited because of severe asphyxia at birth at gestational age 37-40^6^ wks.

### *Substandard factors*

In total, 163 SSF were identified. In 31% of the identified SSF, the guidelines were not followed and in 23% care was not according to normal practice. In 28% the documentation in the patient’s record was insufficient and in 13% communication between caregivers was insufficient or absent. Other SSF were identified in 4% (Table 3). Table 4 shows examples in the largest subgroups of identified SSF and in the subgroups which were considered to address important issues.

**Table 3:** Substandard factors divided into categories and subcategories

**SSF**	**n**	**(%)**	**subgroup**	**n**	**(%)**
Use of guidelines	51	(31)	Delay	8	(16)
			Incomplete use	9	(18)
			Inappropriate use	1	(2)
			Not used, without stating the reason	33	(65)
Normal practice	37	(23)	Delay	6	(16)
			Incomplete use	14	(38)
			Inappropriate use	1	(3)
			Not used, without stating the reason	12	(32)
			other	4	(11)
Documentation	*46	(28)	Base-line data	30	(65)
			Considerations/management	11	(24)
			Delay in correspondence	1	(2)
Communication	*22	(13)	Same echelon, same level	8	(36)
			Same echelon, different level	1	(5)
			Different echelons	8	(36)
			Towards patient	2	(9)
			Between departments	1	(5)
			Other	1	(5)
Other	7	(4)	Medication, tests/investigations, content guidelines	8	(4)
Total	163			158	

* only division in main categories documentation and communication possible. Not enough information to divide into subcategories in 4 and 1 SSF respectively.

**Table 4:** Examples in the largest subgroups of identified SSF which were considered to address important issues

**SSF**	**Subcategory**	**Examples (caregivers involved)**
Use of guidelines	Not used, without stating the reason	-Evaluation of suspected Intra Uterine Growth Restriction (IM) *
		-Post-mortem examinations (G)*
		-Post-partum bladder care (N) *
		-Fentanyl administration (A) *
		-Postnatal paediatric consult when child lives > 1 hr after induction for congenital anomaly (G) *
		-Rectal temperature measurement after axillary measured temperature > 37.5 °C (N) *
Normal practice	Incomplete use	-History taking (IM) *
		-Insufficient time for good care during labour (N) *
		-Follow-up cease-smoking-advice (IM) *
	Not used, without stating the reason	-Evaluation of polyhydramnios (G) *
		-Admission to ICU of critically ill patient (G) *
		-Care management program for patient with borderline personality disorder (IM) *
		-Interval between antenatal visits longer than advised (G) *
Documentation	Base-line data not in patient record	-Base line data on folic acid use, height, weight, ethnic background (IM,G) *
		-Results laboratory tests and ultrasound investigations (M, G) *
	Considerations/management not in patient record	-Decision to perform a Caesarean Section (G) *
		-Choice for particular medication (G) *
Communication	Insufficient within the same echelon and equal professional level	-Handover of maternity care from general practitioner to independent midwife (GP) *
		-Information on the management of a urinary tract infection from general practitioner to independent midwife (GP) *
		-Exchange of patient information between the obstetric, genetics and pathology departments (G,CG,Pa) *
	Insufficient between echelons	-Information from medical specialist to GP and IM after referral mother or child (G) *
		-Conflicting interpretations of post-mortem examination in patient letters to general practitioner and independent midwife (Pa) *

* G,P,M,N,A,CG,GP,Pa,IM = respectievelijk: gaecologist, pediatrician, midwife, nurse, anaesthetist, clinical geneticist, general practitioner, pathologist, independent midwife.

### *Actions*

The analysis of SSF led to 442 actions to improve care. These actions were mentioned in a semi-structured questionnaire defining the categories as described in this section. In the category ‘external cooperation’ (15% of SSF), discussion on a specific subject was taken to another meeting in the cooperation unit dealing with practical matters in day-to-day practice. In the category ‘internal cooperation’ (17%), the actions were mostly related to hand-over situations. In the category ‘practice organization’ (26%), actions were related to medical, organizational and management issues. In the category ‘training and education’ (10%), the training of nurses as well as of gynecologists required action points. In the category ‘medical’ (27%), actions were related to guidelines and normal practice. In the category ‘other’ (7%), peer review and reflection on professional performance were addressed. Table 5 shows examples of actions to improve care by category.

**Table 5:** Frequencies and examples of actions to improve care after perinatal audit meetings divided over categories

**Category actions**	**n**	**(%)**	**Examples (involved caregiver group)**
External collaboration	64	(15)	- Formalising the agreement on the management of reduced fetal movements in local guidelines (G,P)*
			- Strengthening and formalising of informal agreements between 1st and 2nd echelon (G,M)*
			- Strengthening and formalising of informal agreements between specialists 2^nd^ echelon (G,P+A)*
Internal collaboration	76	(17)	- Better and more “to the point” documentation (M)*
			- Clear and specific handover of the care management plan (M+G)*
			- Regular review of all pregnant women in care in the independent practice (M)*
			- Clearer agreement between nurses and doctors on care management plan and communication (G+M)*
Practice organisation	11	(26)	- New routine for updating guidelines and protocols (G+M)*
			- Organisation of better access to guidelines and protocols (M)*
			- Acquisition of a standard reanimation table in the OR (G+P)*
			- Improvement of the procedure for the follow up of laboratory results (M)*
Training and education	42	(10)	- Skills en drills training program (G)*
			- Regular multidisciplinary patient reviews (G+M)*
			- CTG interpretation training for obstetric nurses (N)*
Medical	117	(27)	- Updating and revision of local guidelines (M,G)*
			- Making a standard questionnaire to be used as a guide for the intake consult (M)*
Other	29	(7)	- More peer review within the practice and professional group within the hospital (M,G)*
			- Participating in peer review sessions outside the practice (M)*
			- Taking more time to reflect on ones own professional practice (M,G)*

* G,P,M,N,A=: gynaecologist, paediatrician, midwife, nurse, anaesthetist respectively.

### *Remarks*

1026 questionnaires were filled in after 64 audit meetings. In 77% the open question on the most valued aspects of the meetings was answered. The initial training and the methods for analyzing the SSF were valued most, by 10% and 12% of the respondents, respectively. Furthermore, the multidisciplinary character (13%) and the collective and non-judgmental search (21%) for substandard factors, the perception of safety (13%), facilitated by the training, and the structure of the meetings were also mentioned. The latter enabled the participants to have the confidence to discuss their own care and the circumstances related to the occurrence of a SSF. In many units, this led to an improvement of the cooperation between caregiver groups. Participants found that the meetings motivated them to reflect on their own professional performance (5%) and that they were of postgraduate educational value (10%).

### *Confidential committee*

No participant contacted the confidence committee during the project.

## Discussion

The main finding in this study is that, with our implementation strategy, we succeeded in implementing unit-based perinatal mortality audit meetings in all 15 perinatal cooperation units in the northern region of the Netherlands.

The successful introduction of innovations in healthcare depends on a variety of factors related to the socio-political context, to the organization in which the care is given, to the healthcare professionals themselves, to the innovation itself, and to the facilities needed to implement the innovation [17-19]. Grol et al. found that none of the popular models for improvement of care are superior and that different models should be integrated to achieve change [20]. Knowledge of the field in which a new technology needs to be implemented and awareness of the stages of change necessary to implement and sustain the implementation of the new technology is therefore an essential factor in the whole process [8].

### *Facilitating factors*

Our implementation strategy addressed several issues, which we expected might impede the introduction of a perinatal audit. Firstly, an adverse outcome of pregnancy has an impact on the involved caregivers, which may influence their position within their care providing group, the subsequent care they provide, and their relationship with the patient [21]. By addressing this issue in the training sessions of the audit groups, we created an awareness of the impact of an adverse outcome on the caregivers involved as well as about their reactions in these situations. This facilitated an empathic and safe environment during the audit meetings. In addition, having an independent chairperson for the meetings strengthened the perception of safety in the audit groups.

Secondly, the preparatory interviews provided the project team with local information on the impeding factors to be addressed and the stimulating factors to be used in each cooperation unit [19]. These interviews also proved to be a valuable tool for establishing a low threshold for communication between the core groups and the project team, which created opportunities for offering support. Thirdly, similar to the findings of Belizaán et al., the presence of the members of the project team at all the introductory audit meetings and their non-judgmental participation in subsequent meetings was greatly appreciated by the local caregivers and increased the acceptance of holding a perinatal audit. It also emphasized the importance of a unit-based perinatal audit in all groups and stimulated continuation of the audit meetings [8].

### *Impeding factors*

During the project, a potentially impeding factor for implementing the perinatal audit became apparent. Measures to improve the quality of care, such as perinatal audit, are mandatory in all healthcare facilities, however, the funds for quality improvement activities are limited [22]. Organizing and preparing the audit meetings and holding the meetings was time-consuming and therefore expensive. Consequently, continuation of perinatal audits depends largely on the motivation of the caregivers to reflect on their own care. This motivation will undoubtedly be influenced by the results of the audits and the improvements achieved in providing care.

### *Actions after audit*

Perinatal audit is a cyclic process in which the results of the audit process are translated into healthcare changes, which in turn are subjected to evaluation [23]. Unit-based audits by the caregivers creates the opportunity for taking immediate action after identifying and analyzing substandard factors. The methods for the analysis and the actions to improve care were incorporated in the implementation strategy. During the implementation period, 442 actions were reported. They were, however, not always specific enough to be implemented in the cooperation unit as a whole. This has recently been described by Pattinson et al. and should to be addressed specifically in subsequent programs [24].

### *Additional findings*

Among many other things, good cooperation between caregiver groups and care providers individually is a prerequisite for providing optimal care [25]. According to the participants of the unit-based audit meetings, this cooperation is apparently not always good enough. This was illustrated by a spontaneous report in the questionnaire of the improvement experienced in the relationship between caregiver groups and hospital departments. In addition, the educational value was found to be an important aspect of the audit meetings. Both cooperation and postgraduate education are considered to be indirectly beneficial for the quality of perinatal care [26].

### *International perspective*

National perinatal audit organizations have been established in several western European countries [27,28]. In these countries care is usually evaluated in external audits and the results are fed back to the caregivers. Amelink et al. found that, in the Netherlands, caregivers wish to evaluate their own care and can do so effectively. In fact, their opinions on the quality of their care is sometimes more stringent than those of the external evaluators [29]. It can also be argued that unit-based perinatal audits may be more effective than external audits. In a unit-based audit, feedback is an inherent part of the audit process and the analysis and actions to improve care is considered to be the responsibility of the audit group. Although there is still room for improvement in this process, we found that the audit groups do act on their own proposals for improving the quality of care in their organization.

### *National perspective*

After the implementation of unit-based perinatal audit in the northern region of the Netherlands, our implementation strategy and audit methods are now being implemented nationwide in all 93 units by the National Bureau for Perinatal Audit (http://www.perinataleaudit.nl). In the national implementation strategy, the analysis of the SSF and the follow-up on actions is emphasized. Regional perinatal audit teams, trained by members of the northern project team, are formed to train and support local audit groups and chair the local audit meetings. The first annual report on the national perinatal audit has recently been presented to the Dutch Minister of Health, Welfare and Sport [30].

## Conclusions

Using our implementation strategy, comprising an information plan, a training plan, and an organization plan, we successfully implemented perinatal mortality audits in 15 perinatal cooperation units in the northern region of the Netherlands. Important features embedded in the implementation strategy are the emphasis on the delicate character of the caregivers’ evaluation of their own care and the communication with the core groups in the cooperation units. The balance between costs and benefits of perinatal mortality audit is still a point of concern, as are the actions needed to improve the quality of care after a perinatal audit [30].

## Competing interests

The authors have no potential conflicts of interest to disclose.

## Authors' contributions

MvD, KBe, KBo NvE, DS, WV, LU and JJE planned and designed the study. MvD coordinated the study, analysed the data and drafted the paper. All authors contributed to the interpretation of the data, revision of the content and gave input and participated at all stages of the study. All authors gave their approval to the final version.

## Funding

This project was funded by the Netherlands Organisation for Health Research and Development (ZonMW 94517307).

## Pre-publication history

The pre-publication history for this paper can be accessed here:

http://www.biomedcentral.com/1472-6963/12/195/prepub
